# *Lespedeza bicolor* Extract Ameliorated Renal Inflammation by Regulation of NLRP3 Inflammasome-Associated Hyperinflammation in Type 2 Diabetic Mice

**DOI:** 10.3390/antiox9020148

**Published:** 2020-02-10

**Authors:** Ji Eun Park, Heaji Lee, Sun Yeou Kim, Yunsook Lim

**Affiliations:** 1 Department of Food and Nutrition, Kyung Hee Univerity, 26 Kyung Hee-Daero, Dongdamun-Gu, Seoul 02447, Korea; gh1003@khu.ac.kr (J.E.P.); ji3743@khu.ac.kr (H.L.); 2 Gachon Institute of Pharmaceutical Science, Gachon University, #191, Hambakmoero, Yeonsu-gu, Incheon 21936, Korea; sunnykim@gachon.ac.kr

**Keywords:** *Lespedeza bicolor*, type 2 diabetes, renal inflammation, NLRP3 inflammasome, energy metabolism, oxidative stress

## Abstract

Type 2 diabetes mellitus (T2DM) is a chronic metabolic disorder characterized by hyperglycemia. The chronic hyperglycemic condition causes hyperinflammation via activation of nucleotide-binding oligomerization domain-like pyrin domain containing receptor 3 (NLRP3) inflammasome and abnormally leads to morphological and functional changes in kidney. A previous study showed a protective effect of *Lespedeza bicolor* extract (LBE) on endothelial dysfunction induced by methylglyoxal glucotoxicity. We aimed to investigate whether LBE ameliorated renal damage through regulation of NLRP3 inflammasome-dependent hyper-inflammation in T2DM mice. After T2DM induction by a high fat diet and low dose of streptozotocin (30 mg/kg), the mice were administered with different dosages of LBE (100 or 250 mg/kg/day) by gavage for 12 weeks. LBE supplementation ameliorated kidney dysfunction demonstrated by urine albumin-creatinine at a low dose and plasma creatinine, blood urea nitrogen (BUN), and glomerular hypertrophy at a high dose. Furthermore, a high dose of LBE supplementation significantly attenuated renal hyper-inflammation associated with NLRP3 inflammasome and oxidative stress related to nuclear factor erythroid 2-related factor 2 (Nrf-2) in T2DM mice. Meanwhile, a low dose of LBE supplementation up-regulated energy metabolism demonstrated by phosphorylation of adenosine monophosphate kinase (AMPK) and Sirtuin (SIRT)-1 in T2DM mice. In conclusion, the current study suggested that LBE, in particular, at a high dose could be used as a beneficial therapeutic for hyperglycemia-induced renal damage in T2DM.

## 1. Introduction

Diabetes is a critical metabolic syndrome associated with aberrant glucose metabolism, and cause chronic damage and dysfunction of various organs, such as blood vessels, heart, nerves, eyes, liver, and kidneys. In particular, hyperglycemia-induced renal inflammation can develop chronic lesions with histological and functional defects in kidney [1,2].

Hyperglycemia results in overproduction of reactive oxygen species (ROS), which cause oxidative stress in various organs in cases of diabetes [3,4]. Oxidative stress due to uncontrolled blood glucose leads to activation of the nucleotide-binding oligomerization domain-like pyrin domain containing receptor 3 (NLRP3) [5]. Recent studies have shown that activation of NLRP3 inflammasome in renal cells promotes renal inflammation and contributes to chronic kidney damage [6,7]. NLRP3 inflammasome acts as the molecular sensor that responds to dangers such as pathogen-associated molecular patterns (PAMPs) and danger-associated molecular patterns (DAMPs) [6]. In sensing those dangers, NLRP3 can recruit the apoptosis-associated speck-like proteins including caspase recruitment domain (ASC) and pro-caspase-1 [6]. Activated NLRP3 inflammasome allows the activation of interleukin (IL)-1β by cleaved caspase-1, and is involved in inflammatory response [6].

Pro-inflammatory cytokines include tumor necrosis factor-α (TNF-α), which is activated by free radicals involved in proinflammatory signals by binding to TNF-α receptors on tubular cell surfaces [8]. These responses trigger activation of nuclear factor kappa B (NF-κB) by particularly encouraging the phosphorylated IκB (p-IκB), which allows nuclear translocation of NF-κB [9]. NF-κB activation induces inflammatory cytokines including IL-6 and IL-1β, and maximizes inflammatory response [10]. Chemokines including monocyte chemoattractant protein-1 (MCP-1) are also involved in inflammatory response by recruiting active components of inflammatory cells and adhesion molecules including intercellular adhesion molecule 1 (ICAM-1) [11]. The inflammatory mediators are involved in the attachment of leukocytes, which can release proteolytic enzymes leading to renal damage [11,12]. The various changes promote the loss of function, viability, and harmful mutations. Eventually, excessive oxidative stress and chronic inflammation accelerate radical-mediated damage, resulting in cell degradation and renal damage [13,14]. Hence, suppressing the activation of NLRP3 inflammasome and subsequent hyper-inflammatory response would be a therapeutic target strategy for ameliorating renal damage [15,16,17,18].

The adenosine monophosphatekinase (AMPK)/Sirtuin 1 (SIRT1)/ peroxisome proliferator-activated receptor gamma coactivator α (PGC-1α) signaling pathway is also related to renal damage [19]. SIRT1 is known to protect pathogenesis of diabetic nephropathy (DN) along with regulation of mitochondrial biogenesis [19]. In diabetes, p65 acetylation accelerates the transcription activity of the NF-κB complex [10]. However, SIRT1 interacts with the p65 subunit of the NF-κB complex, deacetylates p65, and consequently suppresses NF-κB activation [20]. Previous studies have focused on the fact that SIRT1 suppresses NLRP3 inflammasome activation [20,21]. Moreover, SIRT1 was found to ameliorate podocyte loss and albuminuria by suppressing the expression of claudin-1 in podocytes [20]. SIRT1 also prevented hyperglycema-induced mesangial expansion by intensifying the AMPK-mammalian target of rapamycin (mTOR) signaling pathway [22]. In addition, PGC-1α, a downstream molecule of AMPK/SIRT1 signaling pathway, suppressed ROS overexpression and renal hyper-inflammation [23]. Hence, activation of the AMPK/SIRT1/PGC-1α pathway could be a possible mechanism associated with the therapeutic approach for hyperglycemia-induced renal damage.

*Lespedeza bicolor* (LB), named by American botanist Asa Gray, is a species of warm-season perennial deciduous shrub, which belongs to the genus *Lespedeza* (Leguminosae), and widely grows in the United States, Asia, and Australia [24]. LB has been used traditionally for the treatment of inflammation of the urinary tract, nephritis, and diabetes [25,26]. Recently, some studies have reported that natural compounds have therapeutic effects on various organ damages [27]. LB also contains many compounds such as genistein, quercetin, daidzein, catechin, rutin, luteolin, and naringin [28]. These natural phytochemicals in *Lespedeza bicolor* extract (LBE) have been determined for their antioxidant and anti-inflammatory activities, as well as their blood glucose lowering effect in hyperglycemia [27,28]. In particular, various polyphenols such as genistein, quercetin, and naringin have an antioxidant function—electron donating and ROS scavenging activity. Our previous study showed that LBE attenuated advanced glycation end product (AGE) formation and breakage in addition to endothelial dysfunction, which was triggered by methylglyoxal-induced glucotoxicity in vitro [29,30]. Furthermore, LB attenuated methylglyoxal (MGO)-induced diabetic renal damage in vitro and in vivo [31]. However, effects of LBE on NLRP3 inflammasome-associated hyperinflammation and AMPK/SIRT1/PGC-1α signaling under hyperglycemic condition have not yet been revealed. Therefore, we investigated whether LBE has ameliorating effects on renal damage by suppressing NLRP3 inflammasome-related hyperinflammation and activating AMPK/SIRT1/PGC-1α signaling in type 2 diabetes mellitus (T2DM) mice.

## 2. Materials and Methods

### 2.1. Extraction of Lespedeza bicolor

Aerial parts of LB were obtained from Jayeonchunsa Co. (Damyang, Korea). LBE was extracted with 70% ethanol at room temperature overnight. Then, the extract was filtered, evaporated, and dry frozen. The obtained hydroalcoholic extract of LBE was kept at −20 °C until it was used. The extract was dissolved in distilled water at 25 mg/mL (LL) and 62.5 mg/mL (HL) independently.

### 2.2. Animals and Study Design

Male 4-week-old C57BL/6 mice (*n* = 50) were purchased from Raon Bio (Gyeonggi-do, South Korea) and were housed in 2–3 per cage in a 12 h light/12 h dark cycle under controlled temperature (22 ± 1 °C) and humidity (50 ± 5%). After 1 week for acclimation, mice were randomly grouped into 2 groups: a normal control group (NC; *n* = 10) which was fed a rodent diet (10% kcal fat, Research Diets, New Brunswick, NJ, USA), and a diabetic group (DM; *n* = 30) which was fed with a high-fat-containing rodent diet (40% kcal fat, Research Diets, New Brunswick, NJ, USA). Food and distilled water were supplied ad libitium.

After 4 weeks of diet treatment, diabetic groups were injected twice with streptozotocin (30 mg/kg body weight, Sigma Aldrich, St. Louis, MO, USA) into peritoneum by a 1 week interval in citrate buffer (pH 4.5) to induce T2DM. Simultaneously, the NC mice were injected with only citric acid buffer. Fasting blood glucose (FBG) levels were measured every week from the tail vein using OneTouch Select blood glucometer (LifeScan Inc., Milpitas, CA, USA) until 5 weeks from the last injection. Mice measured at FBG > 140.4 mg/dL (7.8 mmol/L) at least twice were considered as being in a diabetic state. Diabetes was induced in 30 out of 40 mice. The induction protocol of diabetes was in reference to a previous study by Zhang et al. [32].

Mice considered in a diabetic state were divided into three groups, and all groups (*n* = 10 per group) were differently treated as follows: (A) the NC group, 10% kcal control diet-fed non-diabetic mice group, was supplemented with distilled water; (B) the diabetic control (DMC) group, 40% kcal high fat diet (HFD)-fed diabetic mice group, was supplemented with distilled water; (C) the LL group, HFD-fed diabetic mice group, was supplemented with a low dosage of LBE (100mg/kg vody weight (BW)); and (D) the HL group, HFD-fed diabetic mice group, was supplemented with a high dosage of LBE (250mg/kg BW). Distilled water or LBE freshly dissolved in distilled water was administrated by oral gavage every day for 12 weeks, and 10 h fasting blood glucose level from tail vein was monitored once a week during all supplementation.

At the end of the supplementation period, mice were anesthetized by inhalation with diethyl ether (Duksan, Seoul, Korea). The blood samples were collected by cardiac puncture in heparin-coated tubes (Sigma Aldrich, St. Louis, MO, USA), and were centrifuged at 850× *g* at 4 °C for 15 min to obtain plasma. The kidney was removed from each mouse, weighed, and washed by saline. The kidney tissues were frozen in liquid nitrogen, and were stored at −80 °C before the experiment. Other portions of the kidney were fixed with 10% formaldehyde for paraffin embedding. All experiment protocol was approved by the Institutional Animal Care and Use Committee of Kyung Hee University (KHUASP(SE)-19-076 on 06/14/2019).

### 2.3. Hemoglobin A1c (HbA1c)

HbA1c levels were measured according to commercial reagent methods (Crystal Chem., Downers, Grove, Elk Grove Village, IL, USA).

### 2.4. Renal Function Test

Renal function was examined by measurement of urinary albumin/creatinine ratio (ACR), plasma creatinine, and blood urea nitrogen (BUN). To measure the degree of urinary albumin excretion, spot urine samples were collected by bladder massage at the initial (0–4 week), mid (4–8 week), and late (8–12 week) stages of the experiment. Urinary albumin excretion was determined according to bromocresol green (BCG) albumin quantification method using a commercial albumin assay kit (Bioassay, Hayward, CA, USA). Quantitative urinary creatinine and plasma creatinine levels were measured by Jaffe method [33]. Levels of BUN were examined using a commercial BUN assay kit (Asan pharmaceutical, Seoul, South Korea) according to the manufacturer’s instructions.

### 2.5. Histological Analysis

Kidney was isolated, fixed in 10% formaldehyde solution, dehydrated, and then embedded in paraffin. Sections of renal tissues were cut into 5 μm thickness and stained with hematoxylin and eosin (H&E) through removal of paraffin in xylene and rehydration in alcohol, as per concentration of the series. The stained tissues on slide glass were mounted with histological mounting medium (Histomount, Atlanta, GA, USA) after drying. All images were taken using an optical microscope (Nikon ECLIPSE Ci, Nikon Instrument, Tokyo, Japan).

### 2.6. Protein Extraction and Western Blot Analysis

The kidneys were homogenized in the hypotonic lysis buffer (1.5 mM MgCl_2_, 10 mM 4-(2-hydroxyethyl)-1-piperazineethanesulfonic acid (HEPES), 10 mM KCl, 0.05% nonidet P-40 (NP40), 0.5 mM dithiothreitol (DTT), and distilled water) with protease and phosphotease inhibitor (Thermo Fisher, Waltham, MA, USA), shacked on ice for 1 h, and centrifuged at 1945× *g* at 4 °C for 10 min. The supernatants were centrifuged again at 9078× *g* at 4 °C for 30 min and final supernatants were used as a cytosol extract for Western blot analysis. The remaining pellets were re-homogenized in hypertonic lysis buffer (1.5 mM MgCl_2_, 5 mM HEPES, 0.5 mM DTT, 0.2 mM EDTA, 26% glycerol, and distilled water) with 4.6 M NaCl on ice. After shaking on ice for 1 h, the homogenates were centrifuged at 9078× *g* at 4 °C for 20 min. Then, supernatants were used as nuclear extract for Western blot analysis. Total protein amount of the extract was quantified by bovine serum albumin (BCA) protein assay (ThermoFisher Scientific, Grand Island, NY, USA).

Protein samples were separated with SDS-PAGE and transferred onto poly-vinylidine fluoride (PVDF) membranes (Millipore, Marlborough, MA, USA). After blocking in 3% bovine serum albumin (BSA) in phosphate-buffed saline–0.1% Tween 20 (PBS-T), the membranes were incubated at 4 °C with primary antibodies. Then, the membranes were washed with PBS-T and incubated with respective horseradish peroxide (HRP)-conjugated secondary antibodies for 1 h, and then washed with PBS-T again. The chemiluminescent signals were developed using ECL solution (Bio-rad, Hercules, CA, USA). Images of the developed bands were recorded and quantified with the Syngene G box (Syngene, Cambridge, UK).

### 2.7. Statistical Analysis

Data were expressed as mean ± standard error of the mean (S.E.M.). Statistical significance of differences existed within the experiment. Experimental groups were examined by one-way analysis of variance (ANOVA) using SPSS (version 23.0 for Windows, SPSS Inc., Chicago, IL, USA). Post-hoc analysis was used to identify the differences among the experimental groups at *p* < 0.05 and the corresponding ethical approval code.

## 3. Results

### 3.1. Effect of LBE Supplementation on Body Weight, Food Intake, Fasting Blood Glucose (FBG), and Glycated Hemoglobin (HbA1c) in T2DM Mice

The body weight of the DMC group was significantly increased compared to those of the NC group (Table 1). Simultaneously, there was no effect on body weight change among the DM groups. FBG of the DMC group showed significant elevation compared to that of the NC group, but the LL group showed lower FBG compared to the DMC group (Table 1). Levels of HbA1c in the DMC group were significantly higher than those in the NC group. However, the LL and the HL groups showed lower levels of HbA1c compared to the DMC group.

### 3.2. Effect of LBE Supplementation on Renal Function and Renal Morphology in T2DM Mice

**Figure 1:**
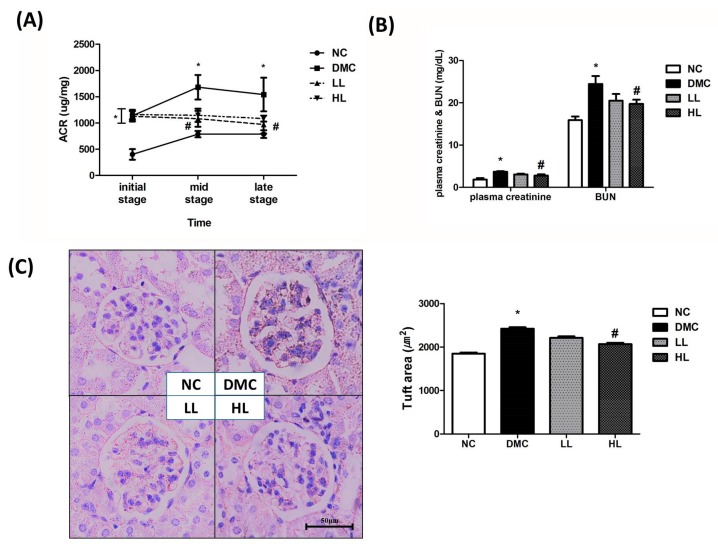
Effect of LBE supplementation on renal function and morphology in T2DM mice. (**A**) Urine albumin/creatinine ratio (ACR) during experiment period, (**B**) plasma creatinine and blood urea nitrogen (BUN), (**C**) kidney morphology (magnification ×400), and glomeruli size. * *p* < 0.05 compared with NC group; # *p* < 0.05 compared with the DMC group.

The ACR in the DMC group was significantly higher than that in the NC group over the whole experiment period (Figure 1A). However, the ACR in the LL group was significantly lower than that in the DMC group at the mid and late stages of the experiment. Plasma creatinine and BUN were significantly higher in the DMC group compared to the NC group. At the same time, a high dose of LBE supplementation significantly decreased the level of plasma creatinine and BUN in the diabetic mice.

In the NC group, capsular space was observed as a thin white line (Figure 1C). Capsular space of the DMC group was thickened compared to that of the NC group. However, a high dose of LBE supplementation improved corpuscular architecture and tubular necrosis compared to the DMC group.

### 3.3. Effect of LBE Supplementation on Renal Receptor for Advanced Glycation end Products (RAGE) Formation in T2DM Mice

**Figure 2:**
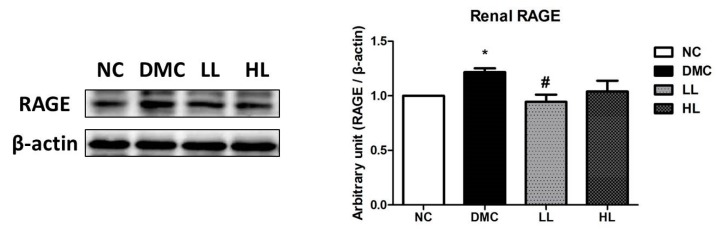
Effect of LBE supplementation on receptor for advanced glycation end products (RAGE) in T2DM mice. A representative band image of repeated experiments is shown in the left panel. * *p* < 0.05 compared with NC group; # *p* < 0.05 compared with the DMC group.

The protein level of RAGE was significantly elevated in the DMC group compared to that of the NC group (Figure 2). The protein level of RAGE was significantly reduced in the LL group compared to that of the DMC group.

### 3.4. Effect of LBE Supplementation on Renal Inflammation in T2DM Mice

**Figure 3:**
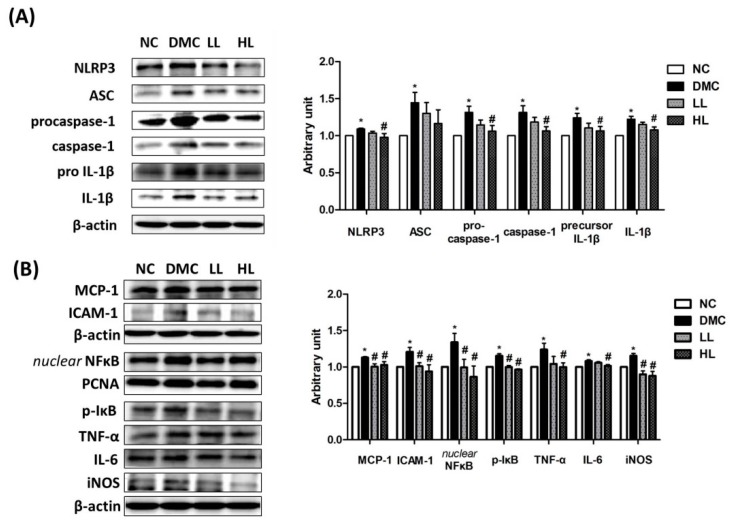
Effect of LBE supplementation on renal NLRP3 inflammasome and inflammation in T2DM mice. Protein levels of (**A**) nucleotide-binding oligomerization domain-like pyrin domain containing receptor 3 (NLRP3) inflammasome: nucleotide-binding oligomerization domain-like pyrin domain containing receptor 3 (NLRP-3); apoptosis-associated speck-like proteins including caspase recruitment domain (ASC), caspase-1, and interleukin (IL)-1β; and (**B**) markers of pro-inflammatory response: monocyte chemoattractant protein-1 (MCP-1) and intercellular adhesion molecule 1 (ICAM-1); and nuclear factor kappa B (NF-κB)-related inflammatory response: nuclear factor kappa B (NF-κB), phosphorylated IκB (p-IκB), tumor necrosis factor-α (TNF-α), interleukin (IL)-6, and inducible nitric oxide synthase (iNOS); representative band images of each marker are shown in the left panel. * *p* < 0.05 compared with NC group; # *p* < 0.05 compared with the DMC group.

The protein levels of NLRP3, ASC, procaspase-1, caspase-1, pro IL-1β, and mature IL-1β were significantly elevated in the DMC group compared to those of the NC group (Figure 3A). However, the protein levels of NLRP3, procaspase-1, caspase-1, pro IL-1β, and mature IL-1β showed significant reduction in the HL group compared to those of the DMC group. There was no significant difference of the protein level of ASC among the DMC group and LBE-supplemented groups.

The DMC group showed greater levels of the protein related to inflammation including MCP-1 and CRP than the NC group (Figure 3B). The DMC group also showed higher protein levels of nuclear NF-κB, phosphorylated IκB, ICAM-1, TNF-α, IL-6, and inducible nitric oxide synthase (iNOS) than the NC group. Simultaneously, the protein levels of MCP-1, nuclear NF-κB, phosphorylated IκB, ICAM-1, and iNOS significantly decreased in both LBE-supplemented groups compared to the DMC group. The protein levels of TNF-α and IL-6 were significantly lowered in the LL group compared to the DMC group.

### 3.5. Effect of LBE Supplementation on Renal Oxidative Stress in T2DM Mice

**Figure 4:**
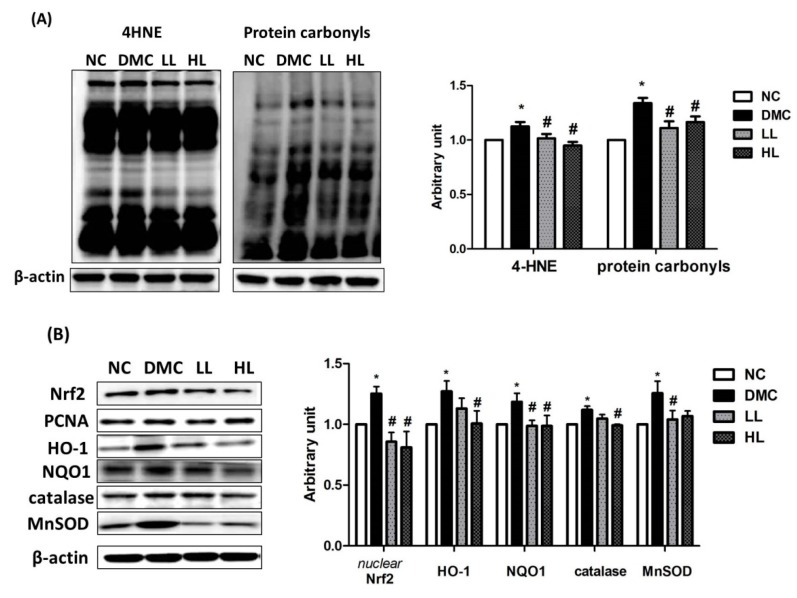
Effect of LBE supplementation on renal oxidative stress in T2DM mice. Representative band images of (**A**) 4-hydroxynonenal (4-HNE) and protein carbonyl groups and (**B**) nuclear factor erythroid 2-related factor 2 (Nrf2)-associated antioxidant defense markers: heme oxygenase-1 (HO-1), NAD(P)H dehydrogenase quinone 1 (NQO1), catalase, and manganese superoxide dismutase (SOD) are shown. * *p* < 0.05 compared with NC group; # *p* < 0.05 compared with the DMC group.

The renal protein level of 4-hydroxynonenal (4-HNE) was significantly higher in the DMC group than that of the NC group (Figure 4A). Simultaneously, the protein level of 4-HNE was significantly reduced in the LBE-supplemented groups compared with that in the DMC group. The level of renal protein carbonyls was significantly increased in the DMC group compared to that in the NC group. However, the level of renal protein carbonyls was significantly decreased by LBE supplementation in the DM group. The protein levels of nuclear Nrf2 (nuclear factor erythroid 2-related factor 2) and cytosolic heme oxygenase-1 (HO-1), NAD(P)H dinucleotide phosphate dehydrogenase quinone 1 (NQO1), catalase, and manganese superoxide dismutase (MnSOD) were significantly higher in the DMC group compared to those in the NC group (Figure 4B). The protein levels of Nrf2 and NQO1 in the LBE supplementation groups were significantly lowered compared to those in the DMC group. The protein levels of HO-1 and catalase in the high dose of LBE supplementation groups were significantly lowered compared to those in the DMC group. The protein level of MnSOD was significantly reduced in the LL group compared to the DMC group.

### 3.6. Effect of LBE Supplementation on AMPK Phosphorylation and SIRT1 in T2DM Mice

**Figure 5:**
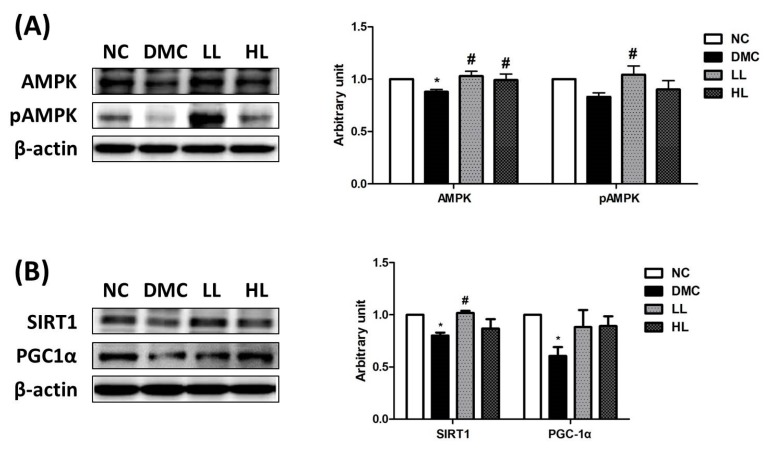
Effect of LBE supplementation on renal adenosine monophosphate kinase (AMPK) phosphorylation and Sirtuin (SIRT)-1/ peroxisome proliferator-activated receptor gamma coactivator-1α (PGC-1α) signaling in T2DM mice. (**A**) Renal AMPK phosphorylation and (**B**) SIRT1-PGC1 activation. Representative band images of each marker are shown in the left panel. * *p* < 0.05 compared with NC group; # *p* < 0.05 compared with the DMC group.

The protein level of AMPK was significantly decreased in the DMC group compared to the NC group and was increased in the LL and the HL groups compared to that of the DMC group (Figure 5A). At the same time, the protein level of phospho adenosine monophosphate kinase (pAMPK) was significantly higher in the LL group compared to that of the DMC group. Consequently, the AMPK phosphorylation ratio (pAMPK/AMPK) was significantly increased in the LL group compared to that of the DMC group.

The renal protein levels of SIRT1 and peroxisome proliferator-activated receptor gamma -coactivator α (PGC1α) were significantly lower in the DMC group than those of the NC group (Figure 5B). Simultaneously, the protein level of SIRT1 was significantly increased in the LL group compared to that of the DMC group. The protein levels of PGC1α were significantly decreased in the DMC group compared to those of the NC group. However, there was no significant difference of the protein levels of PGC1α in the LBE supplementation groups compared to the DMC group.

## 4. Discussion

Various medical plants and natural products have been found to be antidiabetic agents, including banaba, fenugreek, and gymnema, among others. Among these plants, LB is a perennial deciduous shrub belonging to the Leguminosae family and has been used for treatment of inflammation throughout Asia. LB as a legume family contains phenolic compounds including many different types of flavonoid derivatives. The previous study reported by our group found that LBE contained polyphenolic compounds such as quercetin (0.853 mg/g), genistein (0.053 mg/g), daidzein (0.165 mg/g), and naringenin (0.08 mg/g) [24,30,31]. These four components known as major active compounds in Leguminosae have been shown to exert antioxidant effects by inhibiting AGEs formation [24]. Furthermore, quercetin and genistein in LB increased free amine contents, resulting in the increased breakage of AGEs and inhibition of AGE formation [24]. The findings support that the ameliorative effect of LBE against diabetes might be due to synergistic or additive effects of these active ingredients. In the current study, we hypothesized that LBE supplementation could ameliorate hyperglycemia-induced renal inflammation by regulation of NLRP3-inflammasome associated hyperinflammation in an in vivo diabetic model.

First of all, it is well-known that chronic hyperglycemia contributes to renal malfunction such as progression of elevated urinary albumin excretion [34,35]. HFDs induce insulin resistance, and potentially moderate pancreatic beta-cell dysfunction [36]. HbA1c is known to reflect average blood glucose within 3 months. A recent study reported that HbA1c level is a better indicator of hyperglycemia with fewer variables compared to overnight fasting blood glucose [37]. The current study demonstrated that LBE supplementation, regardless of dose, decreased the level of HbA1c in the diabetic mice. Previous studies have shown that decreased glucotoxicity caused by hyperglycemia improved renal function [31,38,39]. In the present study, a high dose of LBE supplementation declined the level of BUN and serum creatinine, and a low dose of LBE supplementation lowered the ACR. Elevated BUN, serum creatinine, and urine ACR level are well-known markers of renal malfunction with albuminuria [40]. Furthermore, the low dose of LBE supplementation attenuated albuminuria and the high dose of LBE supplementation minimized basement membrane thickening in diabetic kidney morphology. It can be inferred that LBE attenuated renal malfunction by regulating hyperglycemia accompanied by morphological alteration of diabetic kidney.

Hyperglycemia-induced oxidative stress results in the formation of AGEs and activation of protein kinase C (PKC). These changes are accelerated by mitochondrial overproduction of ROS in prolonged exposure to hyperglycemia. Activated renal RAGE stimulated production of cytosolic ROS, leading to mitochondrial ROS [41,42]. A previous study reported that LB acts as an AGE modulator by suppressing oxidative stress [30]. Furthermore, LBE ameliorated oxidative stress by reducing RAGE expression, which subsequently reduced AGE–RAGE interaction in MGO-induced renal glucotoxicity [31]. The current study demonstrated that the high dose of LBE supplementation reduced the renal protein level of RAGE in diabetic mice. Both LBE supplementations also suppressed the protein levels of 4-HNE and protein carbonyls. 4-HNE is a representative biomarker of lipid peroxidation, and has cytotoxic and mutagenic activities in various tissues [43,44]. ROS overproduction also leads to oxidative modification of proteins and produces protein carbonyls via lipid peroxidation or glycation/glycoxidation [45]. These data suggest that LBE supplementation ameliorated AGE formation and ROS production by remarkably decreasing lipid peroxidation and protein oxidation. Furthermore, activation of Nrf2 corresponding with 4-HNE and protein carbonyls could serve as an illustration that oxidative stress induces the stimulation of Nrf2 and its related antioxidant defense systems via the compensatory activation against overproduction of ROS. Our results demonstrated that LBE supplementation regulated increased Nrf2 and its related antioxidant defense enzymes in the type 2 diabetic mice.

Moreover, ROS overproduction activates inflammatory response including NLRP3 inflammasome. NLRP3 inflammasome activates various pro-inflammatory cytokines including IL-Iβ, TNF-a, and IL-6. The pro-inflammatory cytokines can critically trigger inflammatory response in diabetic kidney [4,5]. Therefore, suppression of NLRP3 inflammasome could be a potential target of hyperglycemia-induced renal damage [4,5]. A previous study demonstrated that administration of polyphenols inhibited TNF-α-associated pro-inflammatory cytokines [6]. Genistein, quercetin, daidzein, and naringenin in LBE are known to inhibit nuclear NF-κB translocation, along with decreases in iNOS and NO productions in vitro and in vivo [27]. In particular, inhibition of NLRP3 inflammasome could prevent facilitation of the progression of hyperglycemia-induced renal failure [5]. For the first time, the current study showed that the high dose of LBE supplementation suppressed NLRP3 inflammasome along with nuclear NF-κB activation. Indeed, LBE supplementation ameliorated inflammatory responses via suppression of NLRP3 inflammasome.

AMPK has some physiological effects on mitochondrial biogenesis and glucose metabolism. AMPK also ameliorates the NF-κB related inflammatory response through activation of SIRT1 and PGC-1α [9,10]. SIRT1 suppresses NF-κB by deacetylation of p65 and decreases priming of NLRP3 protein [10]. One previous study suggested that SIRT1 decreased activation of NLRP3 inflammasome in vitro [10]. Furthermore, SIRT1 was found to preserve podocyte function by regulating claudin-1, which induces podocyte effacement and albuminuria [46,47]. Hence, the AMPK/SIRT1 signaling pathway could be a therapeutic target in diabetic kidney damage. Other studies suggest that anthocyanin and resveratrol reduce albuminuria, glomerulosclerosis, and tubulointerstitial fibrosis in diabetic nephropathy through the activation of AMPK/SIRT1 signaling [20,21]. The current study showed that LBE supplementation activated SIRT1 and AMPK in diabetic kidney. It would be inferred that LBE supplementation has protective effects on hyperglycemia-induced renal damage by activation of AMPK/SIRT1 along with reduced NLRP3 inflammasome-associated hyper-inflammation in diabetes.

Various pathways accelerate transcription of nuclear NF-κB. Oxidative stress and downregulated AMPK/SIRT1 signaling pathway can directly and indirectly activate NF-κB in hyperglycemia. As mentioned before, our results showed that LBE supplementation has protective action against oxidative stress demonstrated by RAGE, protein carbonyls, and 4-HNE. In particular, the high dose of LBE supplementation was more effective on suppression of NLRP3 inflammasome in our study. However, the low dose of LBE supplementation accelerated the activation of AMPK/SIRT1 more than did the high dose of LBE supplementation in the diabetic condition. The lower protein level of NF-κB in both doses of LBE supplementation might be influenced by various pathways, including AMPK/SIRT1 and oxidative stress as well as NLRP3 inflammasome [48]. In our previous study, LBE supplementation alleviated hepatic inflammation by suppressing the activation of NF-κB. Collectively, LBE supplementation, particularly at a high dose, ameliorates renal inflammation in diabetes according to these findings.

## 5. Conclusions

The current study demonstrated that LBE had protective effects on renal NLRP3 inflammasome-associated hyperinflammation in T2DM mice. Simultaneously, LBE at a high dose stimulated AMPK/SIRT1 activation and attenuated oxidative stress, although some molecular markers were selectively regulated at different treatment doses of LBE in in T2DM mice. Conclusively, the present study suggests that LB might have potential benefit to prevent and ameliorate hyperglycemia-induced renal inflammation under diabetic conditions.

## Figures and Tables

**Table 1:** Effect of *Lespedeza bicolor* extract (LBE) supplementation on body weight, food intake, kidney weight, fasting blood glucose level (FBG), and hemoglobin A1c (HbA1c) in T2DM mice.

Group	NC	DMC	LL	HL
Body Weight (g)				
Before treatment	26.76 ± 0.75	30.94 ± 0.86^*^	31.07 ± 1.01	32.52 ± 1.68
After treatment	30.73 ± 0.61	40.27 ± 2.36^*^	40.27 ± 2.36	40.34 ± 2.59
Gain	3.98 ± 0.33	8.89 ± 0.70^*^	8.09 ± 1.34	7.82 ± 1.72
FBG (mg/dL)	122 ± 7.51	173 ± 14.30^*^	147 ± 9.11^#^	164 ± 10.12
HbA1c (%)	6.62 ± 0.51	9.36 ± 0.15^*^	7.82 ± 0.15^#^	7.12 ± 0.37^#^

All values are means ± SD. * *p* < 0.05 compared with the normal control (NC) group; # *p* < 0.05 compared with the diabetic control (DMC) group. NC, normal mice (negative control); DMC, T2DM mice (positive control); LL, T2DM mice supplemented with low dose (100 mg/kg/day) of LBE; HL, T2DM mice supplemented with high dose (250 mg/kg/day) of LBE.

## Abbreviations

The following abbreviations are used in this manuscript:

ACR	Albumin/creatinine ratio
AGE	Advanced glycation end products
AMPK	AMP-activation kinase
AUC	Area under the curve
BSA	Bovine serum albumin
BUN	Blood urea nitrogen
BW	Body weight
DM	Diabetes mellitus
FBG	Fasting blood glucose
4-HNE	Four-hydroxynonenal
H&E	Hematoxylin and eosin
HO-1	Heme oxygenase-1
ICAM	Intercellular adhesion molecule
IL-1β	Interleukin-1β
IL-6	Interleukin-6
LB	*Lespedeza Bicolor*
MCP	Monocyte chemoattractant protein
NC	Normal control
NF-κB	nuclear factor kappa B
NLRP3	Nucleotide-binding oligomerization domain-like pyrin domain containing receptor 3
NQO1	NAD(P)H dehydrogenase quinone 1
Nrf2	Nuclear factor erythroid 2-related factor 2
SIRT1	Sirtuin1
SOD	Superoxide dismutase
STZ	Streptozotocin
RAGE	Advanced glycation end products receptor
ROS	Reactive oxygen species
T2DM	Type 2 diabetes mellitus
TNF-α	Tumor necrosis factor α
